# A simple six-step guide to National-Scale Hypertension Control Program implementation

**DOI:** 10.1038/s41371-021-00612-6

**Published:** 2021-10-26

**Authors:** Danielle Cazabon, Margaret Farrell, Reena Gupta, Lindsay Joseph, Anupam Khungar Pathni, Swagata Sahoo, Abhishek Kunwar, Kate Elliott, Jennifer Cohn, Thomas R. Frieden, Andrew E. Moran

**Affiliations:** 1 Resolve to Save Lives, an initiative of Vital Strategies, New York, NY USA; 2 University of California San Francisco, San Francisco, CA USA; 3 Resolve to Save Lives, an initiative of Vital Strategies, New Delhi, India; 4 World Health Organization Country Office for India, New Delhi, India; 5 Division of Infectious Diseases, University of Pennsylvania School of Medicine, Philadelphia, PA USA; 6 Columbia University Irving Medical Center, New York, NY USA

**Keywords:** Hypertension, Health care

## Abstract

Hypertension is the leading single preventable risk factor for death worldwide, and most of the disease burden attributed to hypertension weighs on low-and middle-income countries. Effective large-scale public health hypertension control programs are needed to control hypertension globally. National programs can follow six important steps to launch a successful national-scale hypertension control program: establish an administrative structure and survey current resources, select a standard hypertension treatment protocol, ensure supply of medication and blood pressure devices, train health care workers to measure blood pressure and control hypertension, implement an information system for monitoring patients and the program overall, and enroll and monitor patients with phased program expansion. Resolve to Save Lives, an initiative of global public health organization Vital Strategies, and its partners organized these six key steps and materials into a structured, stepwise guide to establish best practices in hypertension program design, launch, maintenance, and scale-up.

## Introduction

Hypertension is the leading single risk factor for death worldwide and accounted for an estimated 10.8 million (9·39–11·5) deaths in 2019 [1]. Hypertension control can be a cost-effective population health “best buy” for countries [2]. Despite this, only 46% of people with hypertension globally are aware that they have the condition, and only 14% of those with hypertension have their blood pressure (BP) controlled [3]. Low- and middle-income countries (LMICs) suffer from a higher total hypertension burden and a much lower BP control rate compared with high-income countries [4].

**Fig. 1:**
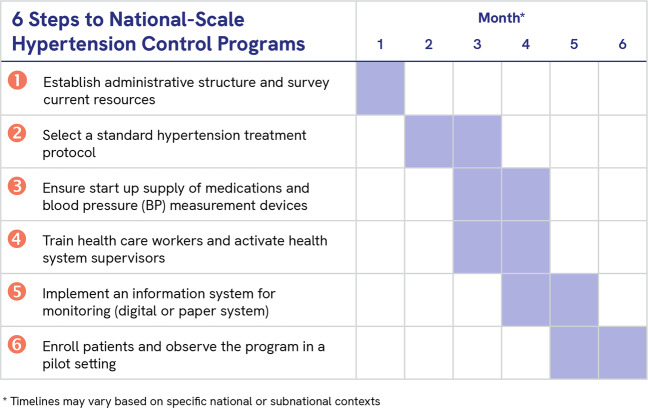
Overview of the Six-Step Guide for National Hypertension Control Programs. The six steps of hypertension control program implementation are presented in chronological order with a sample timeline. The timeline may vary based on specific national or subnational contexts.

To reduce cardiovascular disease burden, the World Health Organization (WHO) and partners developed the HEARTS technical package to guide the management of hypertension and other cardiovascular disease risk factors in resource-limited settings [5]. In 2021, the WHO released new hypertension control guidelines for the first time in 20 years that reinforce the principles of the HEARTS technical package [6]. Since 2017, Resolve to Save Lives, an initiative of global public health organization Vital Strategies, has worked with country governments and other partners to support implementation of HEARTS-based hypertension programs in LMICs. In the India Hypertension Control Initiative, implementation of the HEARTS-based technical package in 24 sites led to a significant increase in BP control across all facility types and age groups [7]. RTSL-supported programs, modeled after the India Hypertension Control Initiative, are also being implemented in 12 countries in the Latin America and the Caribbean region (in partnership with the Pan-American Health Organization (PAHO)) and nine other countries in South Asia, East Asia, Southeast Asia, sub-Saharan Africa, and Europe. As of May 2021, these programs had trained >23,000 health workers to manage hypertension and started over two million people on hypertension treatment, experiences which yielded valuable lessons learned and practical tools and resources. Drawing on the WHO HEARTS package and implementation experiences, Resolve to Save Lives and its partners organized key actions and materials into a structured, stepwise guide to launching large-scale public health hypertension programs. The guide is unique in that it breaks down a hypertension program into six specific steps, where each step includes specific activities (sub-steps) and a corresponding set of practical, standardized tools and resources from various partners which can be adapted to local needs and contexts. The guide includes best practices, tools, and resources from implementation of the Global HEARTS initiative and HEARTS in the Americas [8]. This Six-Step Guide supports country implementers to establish successful large-scale hypertension control programs at the primary health care level in their own countries or subnational areas (Fig. 1) [9]. The resources in this guide are specific to program implementation and are best used after the completion of an initial needs assessment and situational analysis.

## Step 1: Establish an administrative structure and survey current resources

### Steps 1A and 1B: Identify core entities, define roles, and establish a memorandum of understanding and relevant contracts

The first step in implementing a hypertension program is setting up an administrative structure. The administrative structure should include all relevant factors such as national or subnational ministry of health (MOH) focal points. It could also include representatives from the private sector and faith-based health care sector, non-government entities, and civil society.

If the MOH (or an alternative primary implementer) chooses to involve other partners, a memorandum of understanding (MOU) can be established between government entities and partner organization(s) that are collaborating on the hypertension control program (Table 1, Step 1A). Following the signing of the MOU by all parties, where relevant, contracts can be established to legally bind partners to roles and expectations related to the program. Early administrative planning should involve discussions and agreement on hypertension program data storage, security, and access.

**Table 1:** Step 1: Resources for establishing administrative structure and to survey current resources.

Sub-step	Task	Description	General tools and templates
Step 1A	Establish a memorandum of understanding (MOU)	This MOU (or equivalent agreement) is established between partner organization(s) and government entities that are collaborating on the HTN control program.	Memorandum of understanding template [9]
Step 1B	Establish relevant contracts	Contracts can be created to legally bind partners to roles and expectations related to the program and for essential program components such as staffing and workspace.	*There are no tools or templates for this step. Contracts will differ based on partner, vendor type, and country*.
Step 1C	Form a technical working group	A working group will provide technical decision-making.	Guidelines on developing strategic advisory committee [9]
Step 1D	Survey current resources and care delivery models	A baseline survey documents existing staff numbers, antihypertensive medications and BP measurement devices at the national level and the program scope of work. A situation analysis includes workflow planning related to treatment initiation and titration in new patients, along with a differentiated service delivery plan for delivering long-term treatment and retention care for stable controlled patients.	Baseline facility checklist [9] Tool for situation analysis^a^
Step 1E	Budgeting	The budget should be comprehensive, including both product and operational elements for program start-up and maintenance	Program costing tool^a^

^a^Development in progress.

### Step 1C: Form a technical working group

A multi-stakeholder technical working group (TWG) should provide oversight and strategic direction for the program, contribute to technical decisions on programmatic aspects such as site selection, design of a treatment protocol, and establish a process for ongoing program monitoring and quality improvement. The TWG can include representatives from national non-communicable disease programs, supply chain logistics units of the government, health information departments, implementing partners, and civil society or patient representatives. The TWG should survey existing policies and guidance, determine where gaps exist and work with relevant MOH entities to update or create policies and guidance. For example, current government regulations may not support task sharing with nurses or other non-physician health workers for initiation and maintenance of hypertension treatment, a strategy that is feasible and effective [10]. Regulations may need to be updated to support task-sharing and team-based care. The TWG may bring in additional subject matter experts to advise on introduction and scale-up of programs as needed.

### Step 1D: Conduct a needs assessment and survey current resources

Before the program starts, a needs assessment can utilize national and subnational data to better define disease prevalence, current numbers of patients diagnosed and on treatment, relevant national policies, and the regulatory status of key products.

To understand the current level of resources available at primary health care facilities, program staff should conduct a baseline survey at a representative sample of health facilities. Program managers can use the baseline facility checklist to facilitate the documentation of existing human resources, medication and device availability and functionality, lab capacity, the quality of information systems, and how patients are managed (Table 1—Step 1D). These data will inform managers about what resources are needed for the launch of the program.

To ensure long-term hypertension control and patient retention in the program, the baseline situation analysis should also assess opportunities to implement differentiated service delivery as part of the hypertension control program. Differentiated service delivery means less intensive and/or community-based services for hypertension patients with stable controlled BP, freeing up clinic appointments for patients with uncontrolled BP [11].

### Step 1E: Budgeting

Program budgeting should be based on the needs assessment, current resources available, the development of a hypertension treatment protocol (Table 2), and other policies. The budget should be comprehensive, including product, human resources, training, and other operational costs for program start-up and maintenance. The budget should consider existing program funding and document current funding gaps expected with program initiation and expansion and plans to reduce those gaps. When a program is financed by short-term start-up funds, long-term scale-up and sustainability financial planning should commence as early as possible, securing long-term commitments from government and non-government stakeholders.

**Table 2:** Step 2: Resources for selecting a standard hypertension treatment protocol.

Sub-Step	Task	Description	General tools and templates	Country-specific examples
Step 2A	Convene a consensus conference to discuss and agree upon a drug and dose-specific treatment protocol.	Convene the technical working group and any other relevant stakeholders (e.g., MOH officials). Larger countries may opt to establish subnational conferences and protocols, e.g., provincial-level protocols.	WHO HEARTS Evidence-based treatment protocols module [5] WHO tool for the development of a consensus protocol for treatment of HTN [13] Simple, practical HTN treatment protocols (English, Spanish, Chinese) [9]	Ethiopia Hypertension Prevention and Control project Consensus Planning Meeting Agenda [9]
Step 2B	Secure stakeholder approval of protocol	All relevant stakeholders should approve the protocol. Typically, the MOH will have final approval.	*There are no tools and templates for this step. The approval process may vary by institution or Health Ministry*.	
Step 2C	Format and distribute protocol document	The protocol document should have a simple, clear design that lends itself to a poster, job aid or handout. It will be distributed at the facility level for display.	Calcium channel blocker initial monotherapy as first-line treatment [9] Single pill combination as first-line treatment [9]	Treatment protocols: [9] Bangladesh Henan, China Ethiopia Kerala, India Madhya Pradesh, India Punjab, India Nigeria Philippines PAHO countries [29]

## Step 2: Select a standard hypertension treatment protocol

The choice of antihypertensive medications can vary by country, based on the population served, the availability of medications in the region, and medication prices. Simple drug- and dose-specific treatment protocols support efficient and standardized care, enable task sharing, and improve medication adherence [12]. Simple protocols also improve medication demand forecasting, streamline procurement and supply chains, and may lead to medication price reductions by facilitating consolidated bulk procurements of protocol medications [5].

### Step 2A: Convene a consensus conference to discuss and agree upon a drug- and dose-specific protocol

To reach consensus on the drugs, doses, and steps in a simple treatment protocol, convene stakeholders at a consensus conference at the national or subnational level. This allows stakeholders to discuss various protocol options and agree on the best protocol for their country, province, or state [13]. Protocol prototypes should detail specific medication name and dosage at each step, and the schedule for intensifying or adding medications if BP is not controlled. The protocol should be as simple as possible, in linear design with no (or few) branch points to make it easy to for all health worker cadres to follow at the primary health care level.

**Fig. 2:**
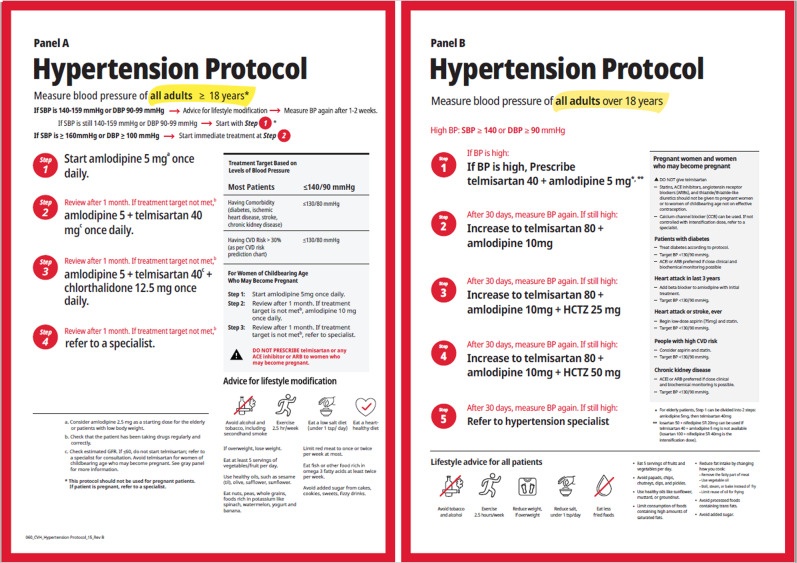
Two examples of simple hypertension treatment protocols for use at the primary care level. Initial single-drug or “monotherapy” protocol (Panel **A**) and initial dual-drug combination therapy protocol (Panel **B**). Note that single pill combinations can be deployed at protocol step 2 (dual-drug combination therapy) and at protocol step 3 (triple-drug combination therapy) in the Panel **A** protocol (initial monotherapy). Dual-drug combination therapy and triple-drug combination therapy can be deployed at steps 1 and 3, respectively, in the Panel **B** protocol (initial dual-drug combination therapy).

The 2021 WHO Guideline for the Pharmacological Treatment of Hypertension in Adults recommends a hypertension treatment protocol that initiates treatment with a calcium channel blocker or a single pill, dual-drug combination of a calcium channel blocker, and angiotensin receptor blocker [6]. Both of these WHO-recommended treatment protocols either introduce sequentially or combine a calcium channel blocker amlodipine, an angiotensin II receptor blocker telmisartan, then, if needed add a thiazide or thiazide-like diuretic. All together, these three medicines—calcium channel blocker, renin-angiotensin system blocker, and thiazide or thiazide-like diuretic—represent the major classes of drugs recommended by experts for effective hypertension control [6, 12]. The specific medications can be replaced by alternate medications at equivalent potency doses from the same drug classes, especially when single pill combination medications are available. For example, if a single pill double- or triple-drug combination (of a calcium channel blocker, angiotensin receptor blocker, with or without a thiazide diuretic) is available in the program setting, one of these can be substituted for the free-dose equivalents of the same drugs (Fig. 2). Where applicable, medicines and BP devices included in guidance should also be included in the country’s essential medicines list and essential diagnostics list.

### Steps 2B and 2C: Secure stakeholder approval, format, and distribute protocol document

Final approval of the treatment protocol is usually given by the MOH. The protocol can then be formatted, printed, and distributed to health care facilities. Facilities can choose to post it on the wall of the room in the primary health care facility where health care workers treat patients and/or distribute it electronically as a job aid.

## Step 3: Ensure start-up supply of medications and BP measurement devices

Medication and BP measurement devices are an essential part of launching a hypertension program. The BP device model chosen for a new hypertension program should be externally validated as accurate, come with multiple cuff sizes, and be durable and affordable for the program [14]. Technical specifications outlined by WHO should be considered when procuring devices [15].

Due to environmental concerns, manual mercury BP devices are being phased out globally [15]. To replace outdated mercury devices, the WHO recommends accuracy-validated automated BP devices over the alternative of manual aneroid BP devices for measuring adult BP. This is because aneroid BP devices require frequent calibration to maintain accurate BP readings and are more prone to observer bias and terminal digit preference [14, 15]. Validated automated digital devices, which inflate automatically and use oscillometric pulses to estimate BP, can be for home or office use, require less skill to operate, eliminate risk of observer bias and terminal digit preference, do not require re-calibration, and require less maintenance [14, 15]. BP devices designed for home use should not be implemented in health care facilities, as they provide accurate readings only up to 30,000 measurements compared to devices designed for office use, which maintain accuracy for up to 100,000 measurements [14]. Automated BP devices purchased for a hypertension program should always be a validated, which means meeting rigorous accuracy standards. National standards for BP devices should be updated to meet WHO-recommended technical standards [15].

### Steps 3A and 3B: Inventory current medications and BP devices

Before forecasting the quantity of medications and BP devices that will be needed for a new hypertension program, program staff should conduct an inventory survey at facility level (Table 3–Step 3A and 3B) to assess quantities of currently available products.

**Table 3:** Step 3: Resources to ensure a start-up supply of medications and blood pressure measurement devices.

Sub-step	Task	Description	General tools and templates	Country-specific examples
Step 3A	Inventory current medications	Conduct an inventory survey. The inventory should include medications at the store and facility level.	Baseline facility checklist [9]	
Step 3B	Inventory current BP devices	Conduct an inventory survey at the facility level.	Baseline facility checklist [9]	
Step 3C	Forecast medication needs	Program supervisors work with facility level managers to project future needs. Initial drug supply forecasting should incorporate program growth scenarios. Forecasting should also plan for multi-month refills (six months or longer) for patients with stable, controlled blood pressure.	Forecasting tool template [9]	Medication forecasting examples [9]
Step 3D	Forecast BP device needs	Program supervisors work with facility-level managers to identify any gaps and project future needs of BP devices.	Blood pressure device forecasting for opportunistic screening [30]	
Step 3E	Procure and monitor medications	Assess the current procurement process and consider alternative options as relevant. Monitor medications on a regular basis and reorder as appropriate. Stock should never fall below a 3-month supply.	Johns Hopkins University Global Hypertension Course (Module 5) [31]	
Step 3F	Procure BP devices	Assess the current procurement process for BP devices and consider alternative options as relevant	How to choose an automated device [9] Selecting BP devices [32] Automated digital BP devices fact sheet [9] WHO technical specifications for automated non-invasive BP measuring devices with cuff [15] Suggested Requirements for External Validation Studies [9] A 90-second primer on automated digital BP monitors [9] Blood pressure measurement device selection in low‐resource settings: Challenges, compromises, and routes to progress [14]	Example of a request for proposal for blood pressure devices [9]
3G	Utilize and strengthen supply chains	Strengthen supply chain for medicine distribution and establish procedures for monitoring and refilling medication inventory.	Drug stock tool—Template [9]	Drug stock tool- Example (India) [9] Min-Max inventory guidance [9, 20] Daily consumption record [9, 20] Ready reckoner job aid [9, 20]

### Steps 3C and 3D: Forecast medication and BP device needs for start up

The Resolve to Save Lives medication forecasting tool can be used to estimate antihypertensive medication requirements for a new hypertension control program (Table 3–Step 3C). The tool is based on the best available program growth and medication consumption assumptions derived from hypertension control program experiences in India and should be adapted to local contexts. Possible modifications include the estimated program drop-out rate, expected patient enrollment, percent of patients requiring treatment escalation at each step or desired coverage in the public sector.

BP measurement device requirements can be calculated based on average daily number of adult patients visiting the facility, the average duration of time to screen one patient, and the number of hours the facility is open each day. Human resource availability also needs to be considered to estimate how many devices are needed for opportunistic screening (Table 3–Step 3D).

### Steps 3E and 3F: Procure medications and BP devices

Program staff should work with national or subnational procurement entities to plan and implement a procurement and distribution strategy for quality-assured medications and BP devices. National procurement entities can source products according to individual national practices, including instituting competitive tendering. Considerations to plan sourcing and procurement include selecting suppliers based on their ability to deliver on-time and in-full, product shelf life at delivery, national registration status, and quality assurance.

The majority of generic manufacturers supply high-quality and affordable antihypertensive medications. Unfortunately, in some cases, antihypertensive medicines have been found to be of poor quality [16] and national programs may want to limit procurement to products with strict regulatory authority approval (for example, United States Food and Drug Administration or European Medicines Agency certification) or manufacturing sites with Good Manufacturing Practice certification by a trusted regulatory body.

At a regional level, pooled procurement mechanisms such as the PAHO Strategic Fund provide a procurement option for Ministries of Health and Government Institutions of PAHO member countries. The PAHO Strategic Fund establishes long-term agreements with manufacturers to offer reduced prices of cardiovascular health medicines and provides technical assistance to strengthen supply management in member counties [17]. Currently, 35 countries and territories are part of the mechanism. A similar approach should be considered in other regions of the world, where variable prices and quality of antihypertensive medications still exist [16].

A number of suppliers provide BP measurement devices meeting WHO-recommended standards. Several reputable hypertension organizations have a catalog of validated BP devices that can be helpful in comparing various models for procurement [18, 19]. Consideration for sourcing should include product warranties, device connectivity, and recommended operating conditions for the given product.

### Step 3G: Utilize and strengthen supply chains to distribute medicines and support monitoring of medication inventory

At a facility level, program staff should inventory the pharmacy to ensure that at least 3 months of medication stock is available to treat the number of current and anticipated new patient enrollments at the facility. The Min–Max inventory guide can be particularly useful when facilities receive stock less than once a month or irregularly (Table 3—Step 3G) [20].

Health facilities should maintain records of receipt and disbursement of all drug stocks. It is recommended that they regularly update the records and report medication status in monthly or quarterly reports to monitor stock levels. Medication records at the facility level can include a stock ledger, which should reflect both stock on hand and transaction history, and a daily consumption record to be utilized where drugs are dispensed (Table 3—Step 3G). As stock ledgers may not be updated regularly, stock verification from registers should be supplemented by the physical counting of available stock. If appropriate and feasible to implement, an electronic logistics management information system can also be helpful to monitor medication inventory. Job aids such as a “ready reckoner” can be prepared to assist facility staff in assessing stock adequacy at a health facility at any point of time (Table 3—Step 3G) [20]. When facility stock falls below a 3-month supply, there should be proactive management to request and distribute additional stock through the supply chain before drug stock decreases to a critical level. Turnaround times from request to distribution should be considered when re-ordering stock.

## Step 4: Train health care workers and activate health system supervisors

### Step 4A: Develop training materials

There are many high-quality training resources on hypertension management, therefore it is best to assemble training materials from pre-existing resources rather than start from scratch. The US Centers for Disease Control and Prevention, PAHO, and Johns Hopkins University have all released courses on hypertension management (Table 4—Step 4A). The India Hypertension Control Initiative training manual is publicly available (Table 4—Step 4A) [20].

**Table 4:** Step 4. Resources to train health care workers and activate health system supervisors.

Sub-step	Task	Description	General tools and templates	Country-specific examples
Step 4A	Develop training materials	When developing materials, consider adapting pre-existing training materials of good quality.	CDC Hypertension Management Training Curriculum [33] PAHO Course: Management of hypertension for primary care team [34] Johns Hopkins University Course: Fundamentals for Implementing a Hypertension Program in Resource-Constrained Settings [31] Training Materials for Simple Application [9, 21] Training materials for implementing differentiated service delivery models for patients with stably controlled hypertension^a^	India Hypertension Control Initiative Training Manual [20]
Step 4B	Train program supervisors	A Training of Trainers program includes individuals such as facility managers and program coordinators, who in turn will train health care workers in their facilities. It is recommended to include a review of program goals as a part of the training.	Training of trainers agenda- Sample template [9]	
Step 4C	Train health care workers	Supervisors facilitate practical training for health care workers.	BP measurement checklist [9] Preparing an individual for BP measurement [32] Why Hypertension is an important issue [32] How to diagnose hypertension [32] What to do after a diagnosis of hypertension [32] Resolve to Save Lives Hypertension FAQs [30] Hypertension differentiated service delivery toolkit^a^	
Step 4D	Establish process for facility-level monitoring and mentorship.	Create a standard form recording intervention fidelity and practice supportive supervision.	Facility checklist – Follow-up visit (card) [9] Supervisory visit facility form [9]	

^a^Development in progress.

### Step 4B: Train program supervisors

Health care worker trainings on hypertension management can be done in a step-down manner, starting with a training of trainers (ToT) session. Facility directors and program managers can participate in a ToT and in turn train health care workers in their respective facilities. Having in-country trainers who can conduct repeated training sessions ensures the training is sustainable.

### Step 4C: Train health care workers

Once trained, supervisors can facilitate practical training for health care workers as part of in-service training. This training covers topics such as measuring BP, hypertension management, data collection, and data entry and reporting. Supervisors should conduct trainings at the facility-level whenever possible to include a wider range of health care workers and reduce disruption to service provision. Training aids have been developed by Resolve to Save Lives to support hypertension control programs (Table 4—Step 4C).

### Step 4D: Establish process for facility-level monitoring and mentorship

Facility-level supervisory visits are helpful for monitoring program quality. Program managers use a standard form to assess and record intervention fidelity, including proper BP measurement, workable equipment, availability of medication, completeness of registers, and data entry practices (Table 4—Step 4D). Ideally site monitoring should be done frequently (monthly or bi-monthly) immediately after a facility is enrolled. Site monitoring and supervision visits are spaced out to quarterly after a site has demonstrated competence. Program managers use results from site monitoring visits to design mentorship or quality improvement interventions.

## Step 5: Information systems (paper-based or electronic)

### Step 5A: Establish hypertension indicators

An information system is crucial to a hypertension program as it facilitates the monitoring of patient and program progress and outcomes. Standard indicators enable programs to compare results across national, subnational, district, and facility levels, and to understand where program quality can be improved [5]. WHO HEARTS and Resolve to Save Lives both provide examples of hypertension program indicators (Table 5—Step 5A). At minimum programs should calculate and report 3–6 monthly cohort measure of hypertension control, cross-sectional control among patients enrolled in the program, loss-to-follow rate up among enrolled patients, and the availability of core antihypertensive medications at the facility level. Ministries of Health and partners should periodically conduct national and/or subnational surveys (e.g., WHO STEPwise approach to surveillance) to assess hypertension prevalence, awareness, treatment, and control and access to essential antihypertensive medications in the overall population.

**Table 5:** Step 5: Resources to implement an information system for monitoring (digital or paper-based information system).

Sub-step	Task	Description	General tool and templates	Country-specific examples
Step 5A	Establish HTN indicators	Establish program indicators based on the HEARTS Systems for monitoring module indicators.	WHO HEARTS Systems for monitoring module [5] Resolve to Save Lives Indicators [9] Differentiated service delivery indicators^a^	
Step 5B	Create portable patient hypertension record	The patient card records patient information, cardiovascular health history, hypertension treatment dates, BP measurements and medications. If information system is digital, include a QR code on the patient card if technology allows.	Paper-based: India Hypertension Control Initiative Simple App digital patient record [9, 21] Digital: India Hypertension Control Initiative non-digital hypertension patient treatment card [9]	
Step 5C	Establish process for monitoring and evaluation	Establish a method for calculating indicators. Plan to examine trends in key indicators and establish feedback loops for quality improvement. Ensure data security and privacy, as well as quality control.	Simplified Indicator Calculator [9] Ten Guiding Principles for Data Collection, Storage, Sharing, and Use to Ensure Security and Confidentiality [35] Standards to Facilitate Data Sharing and Use of Surveillance Data for Public Health Action [35]	Country X Example: Simplified Indicator Calculator [9]
Step 5D	Establish process and timeline for reporting and dissemination of results.	Prepare reports summarizing indicators. Disseminate reports to key stakeholders. Stakeholders could vary by program but may include national or subnational government officials, donors, civil society groups, academic community, patients.	Paper based: India Hypertension Control Initiative registry, annual & quarterly report (non-digital) [9] Digital: Simple application - Hypertension management dashboard [9, 21]	

^a^Development in progress.

### Step 5B: Create portable patient hypertension record

To collect the data needed to calculate hypertension control and monitor program progress, each patient diagnosed with hypertension should have their personal identification and clinical information recorded on a portable hypertension card carried by each patient, in a facility-based longitudinal treatment registry, and as part of a larger registry maintained by local population health managers. If the program has a digital information system, a QR code can be included on the hypertension card to ensure accurate patient tracking if the technology allows (Table 5—Step 5B).

### Step 5C: Establish process for data analysis and use

Monitoring and evaluation officers in countries that have paper systems may find calculating indicators manually a long and tedious process. If a hypertension program does not utilize electronic data, basic digital spreadsheet-based tools can aggregate data from paper patient treatment cards (or a simple random sample thereof; Table 5—Step 5C). Methods of data aggregation should reflect local program conditions and tools can be modified accordingly.

Facility managers, district health officers, and program officers should plan to regularly review data, examine trends in the key indicators, and establish feedback loops. Such regular review of data is critical to help identify program weaknesses and design quality improvement interventions.

It is critical to ensure data security, privacy, and quality control when analyzing patient data. Resources on the security, confidentiality, and appropriate use of data are available from the US Centers for Disease Control and Prevention (Table 5—Step 5C).

### Step 5D: Establish process and timeline for reporting

**Fig. 3:**
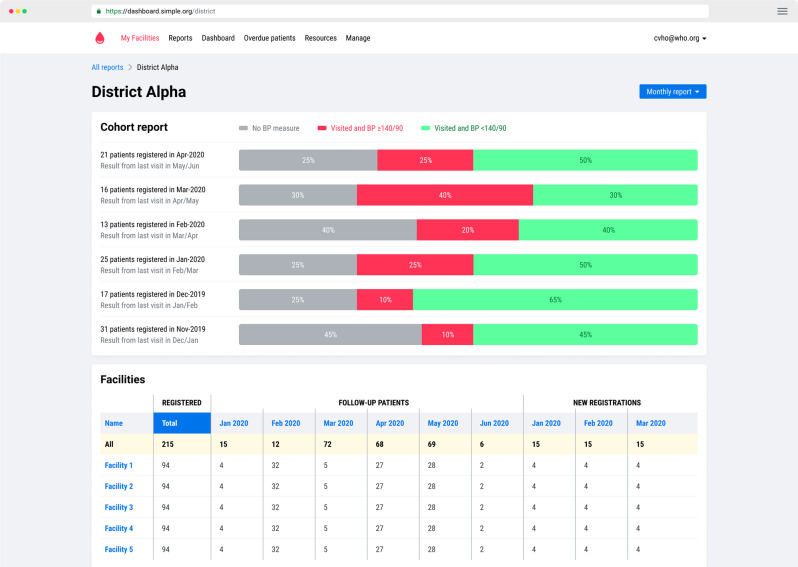
Resolve to Save Lives Simple app hypertension program management dashboard example. Includes control rates, loss-to-follow-up rates, and compares facility performance.

Programs using an electronic data collection system use computer software to automatically calculate program indicators and generate reports. For example, programs using the Resolve to Save Lives *Simple* application, a mobile-based electronic information system designed for hypertension, have access to the Simple app dashboard, which allows program managers to view indicators across districts and over time, and generate monthly, quarterly, or annual reports [21] (Fig. 3; Table 5—Step 5D).

Regardless of how data are collected, monitoring and evaluation officers in facilities should prepare reports based on data aggregated manually, by a digital spreadsheet program, or via an integrated software program. At minimum, reports typically include data on number of patients enrolled, number of patients treated, proportion with controlled BP, and patient follow-up rates. Establishing a process and timeline for reporting will facilitate the analysis and interpretation of program results.

## Step 6: Enroll patients and observe the program in a pilot setting

### Step 6A: Opportunistic screening

An effective way to screen as many patients as possible is to set up opportunistic screening—that is, placing BP devices at registration and in well-trafficked areas, so that all patients entering the facility have their BP measured. In cases where initial screening identifies high BP in the community, care should be taken to make a rigorous BP measurement in a controlled clinic setting to confirm hypertension diagnosis.

When patients are diagnosed and registered, facility staff must record their contact information accurately. This facilitates monitoring treatment and ensuring follow-up.

### Step 6B: Manage existing hypertension patients

Establishing a standardized system for patient follow-up is essential. Patients are lost to follow-up for various reasons, some of which include irregular availability of service providers, side effects of medications, medication stock-outs at the facility, distance to the facility, or preference for the private sector [20]. Strategies for reducing patient loss-to-follow-up include ensuring the availability of medications and service providers, decentralization of services to allow follow-up near the patient’s home or worksite, patient counseling and education, proper documentation of patient contact information so that return-to-clinic reminders can be sent, and documentation of every visit [20]. Other practical lessons can be learned from health system responses to COVID-19 lockdowns in India and Thailand in the year 2020, during which government bodies implemented patient-centered interventions such as community delivery of antihypertensive medication, telemedicine, and multi-month (3- and 6-month) refills to ensure that patients with non-communicable diseases continued to receive care (Table 6—Step 6B) [22, 23]. These practices may be continued after the crisis situation and help reduce the barriers to patients remaining in care.

**Table 6:** Step 6: Resources to guide patient enrollment and observe the program in a pilot setting.

Sub-step	Task	Description	General tools and templates	Country-specific examples
Step 6A	Opportunistic screening	Encourage placement of BP devices in highly trafficked areas of health care facilities so that all patients receive a BP measurement at registration. Establish new patient referral linkages from district hospital facilities to local primary care facilities.		
Step 6B	Manage existing HTN patients	Establish a standardized system for patient follow-up during treatment initiation and for patients with stably controlled BP. Consider implementing a team-based care model, which can alleviate shortages of medical doctors and nurses and allow more decentralized care.	Line-lists and follow-up systems to retain patients in care during treatment initiation^a^ Standard operating procedures for implementing differentiated service delivery models for retaining stably controlled patients in care^a^ WHO HEARTS Team-based care module [24] Task sharing with non-physician health care workers for management of blood pressure in low-income and middle-income countries [10]	Innovations to Sustain Non-Communicable Disease Services in the Context of COVID-19: Report from Pakkred District, Nonthaburi Province, Thailand [22] Community drug distribution at doorsteps: Essential health services decentralized to care for hypertensives under the India Hypertension Control Initiative [23]
Step 6C	Community-based screening and management	Identify well-trafficked locations or events in the community where community health care workers can conduct screening. Establish new patient referral linkages and lost-to-follow-up patient retrieval process from community to local primary care facilities. Consider house-to-house hypertension screening. Consider delivering hypertension treatment in the community through a differentiated service approach that provides medications and monitoring in the community and fewer clinic visits for stably controlled patients.		Hypertension Control in Integrated HIV and chronic disease clinics in Uganda in the SEARCH study [25]

^a^Development in progress.

Team-based care, including “task-sharing” among health care workers alleviates the shortage of medical doctors and nurses in many countries and allows more decentralized care that is delivered in the community where patients live. Team-based care occurs when all members of a multidisciplinary team engage in a patient’s care. This could involve training new staff to fill gaps in the team or building the capacity of existing team members so they can take on new tasks [24]. Using a team-based care approach can also enable more tasks to take place at primary care level, where facilities are often staffed by nurses and community health workers. Patients requiring specialized care can be referred to higher levels of care and when patients are stable, they can be followed up at lower levels of care. Compared with usual care, a task-sharing approach applied for hypertension management in LMICs led to a mean reduction of systolic BP by 4·85 mm Hg (–6·12 to –3·57) and a mean decrease in diastolic BP of –2·92 mm (–3·75 to –2·09 Hg) (Table 6—Step 6B) [10].

### Step 6C: Community-based screening and management

Patient-centered care can also involve hypertension screening by health care workers in the community—in well-trafficked locations, at events, or even house-to-house. Strong referral linkages must be established from the community to local primary care facilities so that individuals with high BP receive a formal diagnosis and treatment. Other health care programs, such as for diabetes or HIV, may already have established screening or treatment programs, therefore it may be efficient to integrate hypertension services into established practices (Table 6—Step 6C) [25].

## Discussion

This Six-Step Guide and best practice guidance was developed from implementation experience of 21 Resolve to Save Lives-supported national hypertension control programs. Despite successful enrollment and management of patients in these programs, there are continuing challenges to achieving and maintaining population hypertension control. Over time, country programs have developed solutions to address these challenges.

It is essential that each country program integrates their own simple hypertension treatment protocol into national hypertension guidelines as part of national scale-up across all primary health care facilities. The next step globally will be to gather international consensus and converge on a universal simple treatment protocol shared across countries. A global standard hypertension treatment protocol can streamline global medication procurement for a short list of generic quality-assured preferred medications. Consolidating demand for antihypertensive medications across states or countries that are procuring the same preferred medications creates negotiation power with manufacturers which can lead to price reductions. Similar market-shaping interventions have been used successfully in the past to lower prices of medications for malaria and HIV commodities [26]. While this convergence has been challenging, Resolve to Save Lives-supported programs are building the evidence for these market-shaping approaches through demonstration programs.

Achieving hypertension control means utilizing programmatic data to inform program improvements and spark innovations that improve the treatment experience and health of patients. Resolve to Save Lives-supported hypertension control programs leverage individual patient and health care facility-level data to inform systematic quality improvement efforts. Systematic continuous quality improvement identifies program challenges on a regular basis and takes rapid actions to address them. Feedback loops that are integral to the quality improvement process are easier and faster to operate from an electronic health information system but can also be based on data from aggregating paper records on a monthly or quarterly basis.

Lessons learned from the pilot stages of the hypertension program should be reviewed and considered before scaling the hypertension program to new areas. A situational analysis of the expansion areas may also be helpful to tailor the program to local contexts.

A significant challenge that arises in many donor-led projects is how to transition from donor funding to government funding for the program [27]. This has been addressed early in Resolve to Save Lives-supported hypertension programs by implementing the program jointly with the appropriate government departments and utilizing existing infrastructure and MOH staff. This can increase the likelihood of progressive country ownership. Step 1 of the 6-step guide includes initiating advanced planning for a transition to local government and other stakeholder ownership, including financing and implementation of enabling regulations and policies.

Other challenges and solutions have been identified through implementation of these hypertension programs in LMICs (Table 7). Population health gains from the clinical hypertension control approach detailed in this guide can be augmented by additional BP lowering achieved by a complementary population-wide dietary sodium reduction program [28].

**Table 7:** Ongoing challenges in existing hypertension programs and corresponding solutions.

Step	Challenge	Solution
1→ Establishing administrative structure	• Transitioning from donor-funded program to government-owned program.	• Jointly implement program with ministry of health from program start. • Develop a transition plan that considers roles, responsibilities, and budgeting for products and services long-term.
2 → Treatment protocols	• Converging on a universal treatment protocol.	• Build evidence for treatment protocols through demonstration programs.
3→ Medications and BP devices	• Medication stock-outs • Limited uptake of fixed dose combination medications. • Variable medication quality and affordability • Lack of availability of validated BP devices. • Poor awareness among providers and program managers of the importance of BP device validation. • High cost of BP devices.	• Market shaping to reduce prices of fixed dose combination antihypertensive medications. • Strengthen procurement and supply chains in LMICs. • Build capacity of ministry of health staff to forecast medication supply needs. • Advocate for reduced out-of-pocket medication fees for patients • Advocate for coverage of NCD medications and services under national health insurance schemes.
4→ Training of health care workers and supervision	• Learnings from training not being implemented or sustained. • Need for frequent trainings due to frequent turnover of staff.	• Assess the impact of training and areas for improvement. • Conduct refresher training. • Provide ongoing clinical mentorship. • Expand training to include community health workers, patient champions, and community-based providers.
5→ Information systems	• Lack of electronic health records • Limited use of data for program improvement.	• Government investment in electronic health records. • Build capacity for continuous quality improvement utilizing program data. • Include key hypertension control indicators in program reviews at national/subnational levels.
6→ Enroll patients and pilot	• Transitioning from pilot projects to scale-up. • Lack of human resources for scale-up.	• Scale-up team-based care through capacity building of existing health care workers and training new cadres of health care workers. • Roll-out packages of differentiated service delivery specific to location context. • Government investment in hypertension care.

## Conclusion

Starting a large-scale, national public health hypertension control program can be broken down into six practical steps. Resolve to Save Lives’ practical Six-Step Guide to implementing hypertension control programs is a resource for governments and other implementers that establishes best practices in hypertension management and provides a roadmap for program launch, maintenance, and scale-up. The Six-Step Guide distills best practices from the WHO HEARTS technical package and the 2021 WHO guideline for the pharmacological treatment of hypertension in adults. Implementing and scaling up WHO HEARTS-based hypertension control programs in countries around the world will control high BP worldwide, and thereby prevent unnecessary deaths and disability for millions of people.

## Summary

### What is known about this topic

- United Nations Sustainable Development Goal 3 includes reducing premature mortality from non-communicable diseases by one-third.
- Hypertension, a non-communicable condition, is the leading risk factor for death worldwide; most of global hypertension-related disease burden occurs in low- and middle-income countries.
- Large-scale public health hypertension control programs must be prioritized to prevent hypertension-related disease burden in low- and middle-income countries.

### What this study adds

- A practical, stepwise implementation guide to launch national hypertension control programs.
- Practical tools that can be adapted by countries to support hypertension control program implementation.
